# Unlocking ferroptosis heterogeneity: ATF4 versus SREBF transcriptional programs

**DOI:** 10.1038/s41420-026-03238-0

**Published:** 2026-07-03

**Authors:** Mariia V. Barannikova, Valeriy K. Sulyagin, Dmitry A. Korzhenevskii, Varvara D. Starodubova, Maria A. Sorokina, Alexey M. Nesterenko, Guzel R. Gazizova, Victoria P. Sulyagina, Oleg A. Gusev, Elena I. Shagimardanova, Vsevolod V. Belousov, Olga M. Kudryashova, Arina G. Shokhina

**Affiliations:** 1 Federal Center of Brain Research and Neurotechnologies, Federal Medical Biological Agency, 117513 Moscow, Russia; 2 Moscow Center for Advanced Studies, 20, Kulakova Str, 123592 Moscow, Russia; 3 Pirogov Russian National Research Medical University, 117997 Moscow, Russia; 4 Faculty of Biology, Lomonosov Moscow State University, 119234 Moscow, Russia; 5 LIFT, Life Improvement by Future Technologies Institute, 121205 Moscow, Russia; 6 Regulatory Genomics Research Center, Institute of fundamental medicine and biology, Kazan Federal University, Kazan, Russia; 7 Intractable Disease Research Center, Juntendo University, 113-8421, Tokyo, Japan; 8 Genomics and Bioimaging Core Facility, Moscow, 121205 Russia

**Keywords:** Transcriptomics, Proteomics, Stress signalling

## Abstract

Regulated cell death (RCD) encompasses diverse forms induced by common cellular stressors, including oxidative stress and endoplasmic reticulum stress. Cells may display features from multiple RCD types, implying coexistence of “pure” and “mixed” forms of cell death. Ferroptosis is an iron-dependent form of RCD characterized by lipid peroxide accumulation and absence of specific biomarkers. Its regulation is heterogeneous and pathway-dependent, reflecting the broader complexity of the RCD spectrum. This study hypothesized two major regulatory pathways governing ferroptosis induction and functioning. The first pathway operates through GPX4, a central enzyme preventing ferroptosis by reducing lipid peroxides. Genetic GPX4 knockout and chemical GPX4 inhibition with ML210 resulted in significant upregulation of mevalonate pathway regulators SREBF1 and SREBF2, indicating compensatory anti-ferroptotic activation through the mevalonate pathway. The second pathway functions through the Xc⁻/GSH system critical for glutathione synthesis. System Xc⁻ inhibition leads to intracellular cysteine depletion with subsequent glutathione depletion, inducing ferroptotic cell death. SREBF transcription factor cascades were downregulated in system Xc⁻/GSH depletion experiments, contrasting with GPX4 inhibition responses. Instead, the system Xc⁻/GSH pathway is primarily governed by ATF4 to maintain cystine uptake and glutathione biosynthesis under stress conditions. These findings confirm ferroptosis as a heterogeneous spectrum of cell death with multiple interacting yet mechanistically distinct pathways. Moreover, these data provide a dynamic multi-omics resource for further ferroptosis research.

**Figure:**
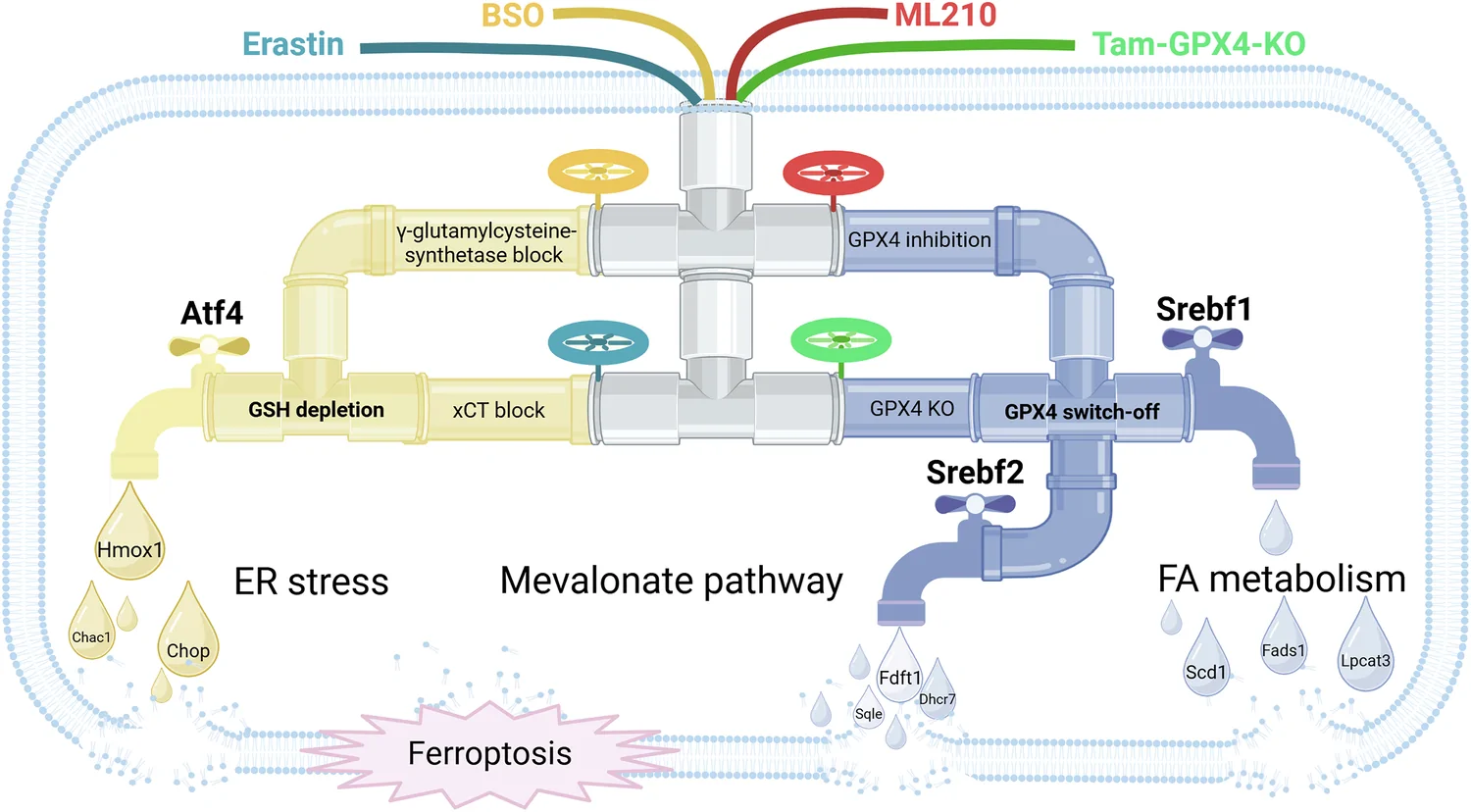

## Introduction

Ferroptosis is a regulated cell death characterized by the iron-dependent accumulation of lipid peroxides, leading to cellular damage and ultimately death [1]. Ferroptosis is characterized by its lack of specific biomarkers and by its phenotypic heterogeneity: recent multi-omics analyses have demonstrated that ferroptotic cells can share transcriptomic and metabolomic features with other regulated cell death programs, suggesting that ferroptosis does not represent a uniform endpoint but rather a spectrum of overlapping cellular states [2].

Cells employ two primary and interconnected mechanisms to protect against ferroptosis. The central axis operates through glutathione peroxidase 4 (GPX4), a critical enzyme that prevents ferroptosis by reducing lipid hydroperoxides to non-toxic lipid alcohols [3]. This process relies on reduced glutathione (GSH), which is synthesized from cysteine derived from extracellular cystine imported via the glutamate/cystine antiporter system xc⁻. Alongside the GPX4/GSH axis, additional cell-intrinsic mechanisms including coenzyme Q-dependent lipid radical scavenging contribute to ferroptosis resistance, and their interplay with the xc⁻/GSH/GPX4 axis constitutes the core regulatory network investigated in the present study [4, 5].

Recent studies emphasize the importance of lipid metabolism and the mevalonate pathway in modulating ferroptosis. The mevalonate (MVA) pathway protects against ferroptosis by producing Coenzyme Q10 (CoQ10), a lipophilic antioxidant that mitigates oxidative stress as a substrate of FSP1 [6], and by regulating the synthesis of GPX4 influencing the maturation of selenocysteine tRNA necessary for GPX4 translation [7]. Moreover, previous studies demonstrated that 7-dehydrocholesterol (7-DHC), one of the MVA by-products, being highly susceptible to oxidation acts as an endogenous suppressor of ferroptosis by shielding phospholipids from autoxidation [8].

Sterol regulatory element-binding proteins (SREBPs), specifically SREBF1 and SREBF2, are the master transcriptional regulators of the mevalonate pathway and lipid metabolism, controlling the expression of genes involved in cholesterol and fatty acid biosynthesis. The incorporation of polyunsaturated fatty acids (PUFAs) into membrane phospholipids increases susceptibility to lipid peroxidation and ferroptosis, whereas monounsaturated fatty acids (MUFAs) can protect by stabilizing membrane integrity [9]. The context-dependent involvement of SREBPs in modulating ferroptosis requires additional investigation.

Endoplasmic reticulum (ER) stress represents another critical regulatory mechanism in ferroptosis through activation of the unfolded protein response (UPR). The UPR consists of three major branches: PERK (protein kinase RNA-like ER kinase), IRE1α (inositol-requiring enzyme 1α), and ATF6 (activating transcription factor 6), which exhibit distinct and sometimes opposing roles in stress-response regulation. The PERK-eIF2α-ATF4 signaling axis demonstrates a dual function in ferroptosis: ATF4 can protect against ferroptosis by transcriptionally activating SLC7A11 to maintain glutathione biosynthesis and cystine uptake under stress conditions [10]. Conversely, IRE1α promotes ferroptosis sensitivity through its endoribonuclease activity, which directly cleaves and downregulates mRNAs encoding glutathione biosynthesis regulators GCLC and SLC7A11, independent of its canonical UPR function [11]. This intricate relationship between ER stress pathways and ferroptosis regulation adds another layer of complexity to cellular death-survival decisions and highlights the context-dependent nature of these stress response mechanisms.

Despite the considerable advances in understanding ferroptosis regulation through individual pathways, a comprehensive comparative analysis of how different ferroptosis induction mechanisms activate distinct cellular responses has remained unexplored. While previous studies have characterized ferroptosis through the lens of single pathways or specific molecular targets, the potential for pathway-specific transcriptional programs and their differential impact on cellular antioxidant defense systems has not been systematically investigated. Here, we present the first comparative multi-omics analysis of ferroptosis induction mechanisms, revealing that different approaches to triggering ferroptosis activate fundamentally distinct transcriptional programs that diverge in their cellular stress responses, antioxidant mobilization patterns, and metabolic reprogramming strategies.

## Results

### Temporal dynamics of ferroptosis induction across multiple targeting strategies

**Fig. 1:**
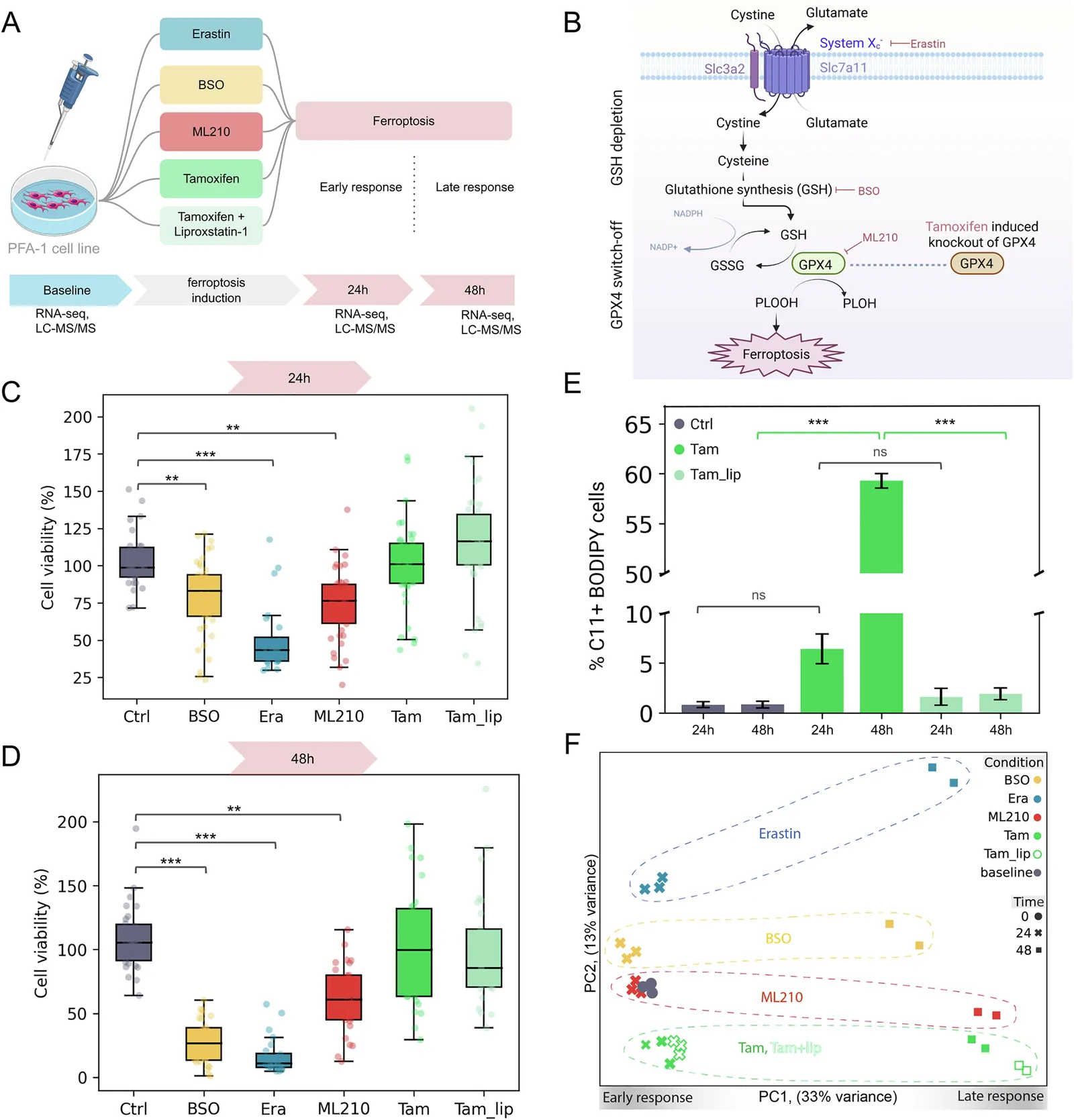
Study design, ferroptosis validation and data structure. **А** Schematic representation of the experimental pipeline. Chemical ferroptosis inductors: erastin (Era), L-buthionine sulfoximine (BSO), ML210. Genetically induced ferroptosis: tamoxifen-induced GPX4 knockout with or without ferroptosis inhibitor liproxstatin-1 (LIP-1). All conditions were analysed at two time points after ferroptosis induction: early (24 h) and late (48 h) by RNA high-throughput sequencing and proteome sequencing by the liquid chromatography–mass spectrometry (LC-MS/MS). **B** Ferroptosis inductors used in the study pipeline. BSO inhibited γ-glutamylcysteine synthetase, Era blocked cystine–glutamate antiporter, ML210 directly inactivated GPX4, Tamoxifen was used to knockout GPX4, LIP-1 was used to assess the impact of ferroptosis induction on gene expression without ferroptotic death itself. **C, D** Cell viability analysis in PFA1 cells under ferroptosis induction via erastin (Era, 0.5 µM), L-Buthionine-sulfoximine (BSO, 30 µM), ML210 (0.3 µM), (Z)-4-Hydroxytamoxifen (Tam, 1 μM), (Z)-4-Hydroxytamoxifen (1 μM) and Liproxstatin-1 (0.5 µM) (Tam_lip) for 24 h **C** and 48 h **D**. ** indicates *p*‑value < 0.01 and *** indicates p‑value < 0.001. **E** Quantification of lipid peroxide accumulation in the tamoxifen-induced GPX4 knockout model and complete rescue by liproxstatin-1. Bar graph showing the percentage of BODIPY 581/591 C11-positive cells measured by flow cytometry in Pfa1 cells at 24 h and 48 h timepoints. Three conditions are compared: untreated control (Ctrl, gray), tamoxifen-treated cells (Tam, green), and tamoxifen + liproxstatin-1 co-treated cells (Tam_lip, light green). Data are presented as mean ± SEM from n ≥ 3 independent biological replicates. Statistical significance was assessed by Welch’s t-test, ***p < 0.001; ns - not significant. Representative flow cytometry dot plots are shown in Supplementary Fig. 1. **F** Principle component (PC) analysis of the RNA-Seq data. PC1 showed difference between early and late responses, PC2 indicated separation between types of ferroptosis induction.

To investigate ferroptosis molecular mechanisms, we induced ferroptosis by targeting different components of the xc⁻/GSH/GPX4 axis using both genetic (tamoxifen-induced GPX4 knockout) and chemical approaches: erastin (Era) to block system xc⁻, BSO to inhibit glutathione synthesis, and ML210 to directly inactivate GPX4 (Fig. 1A, B). All conditions were analyzed at early (24 h) and late (48 h) timepoints using RNA-seq and total proteomics.

Ferroptosis induction was confirmed across chemical induction conditions by significant reductions in cell viability at 24 h and 48 h (Fig. 1C, D). Tamoxifen-treated cells did not show significant MTT viability changes within the 48 h observation window, consistent with the gradual kinetics of GPX4 protein depletion in this inducible knockout system. To validate the ferroptosis specificity of this response, co-treatment with liproxstatin-1 (LIP-1), a potent ferroptosis inhibitor, was performed in the Tam model as a proof-of-concept rescue experiment. LIP-1 co-treatment completely prevented lipid peroxide accumulation at both timepoints (Fig. 1E, Supplementary Fig. 1), consistent with the well-established ferroptosis-suppressive activity of this compound.

Principal component analysis of RNA-seq data validated our experimental design: samples clustered primarily by timepoint (PC1, 33% variance) and secondarily by induction method (PC2, 13% variance) (Fig. 1F). The close clustering of Tam and Tam+LIP-1 samples indicated that while liproxstatin-1 effectively prevents ferroptotic lipid peroxidation, it does not reverse the transcriptional stress response associated with GPX4 loss.

### Deferrinda - differential expression ferroptosis induction atlas

**Fig. 2:**
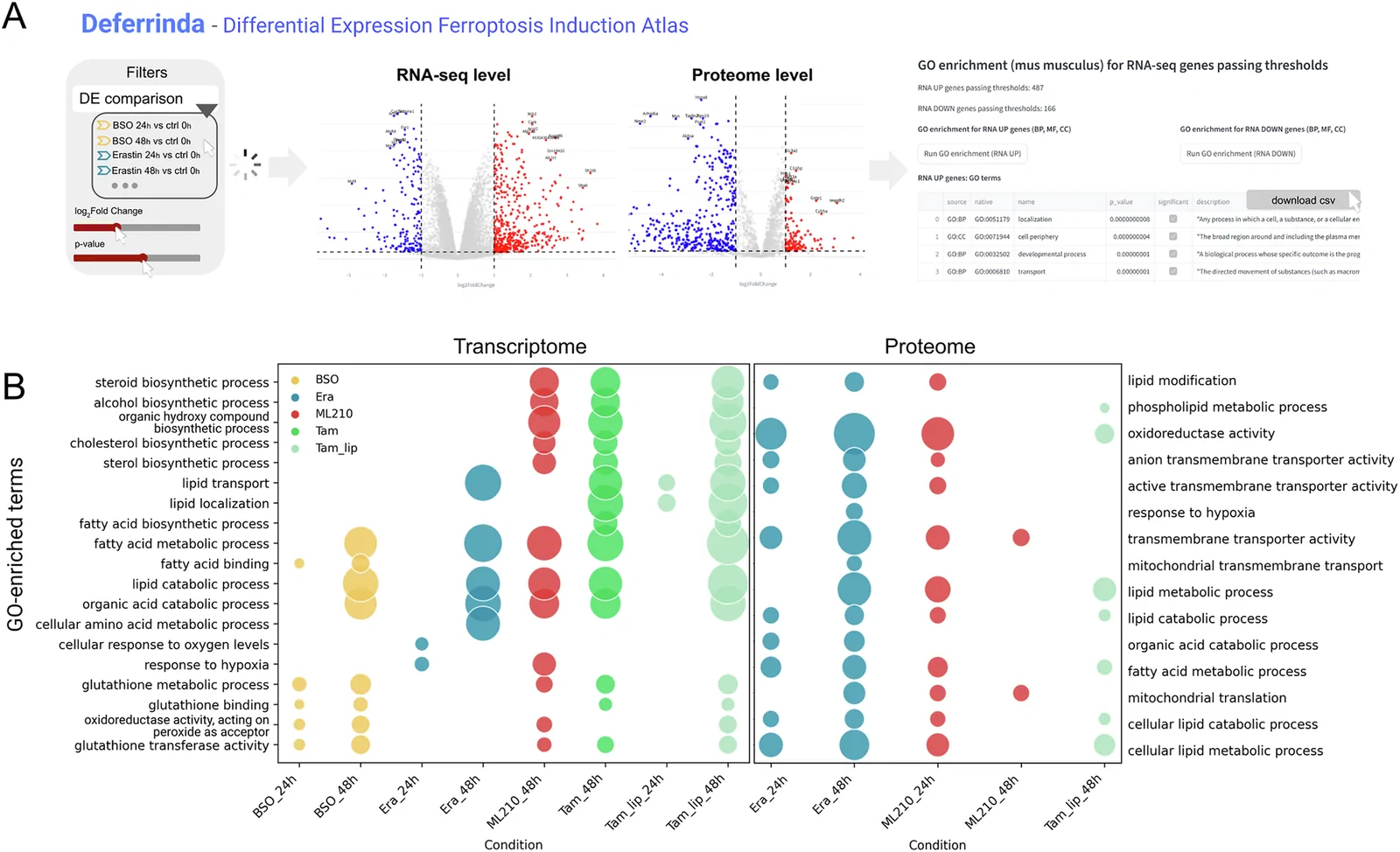
Deferrinda database interface and functional enrichment analysis of ferroptosis-induced transcriptome and proteome changes. **A** Schematic overview of Deferrinda (Differential Expression Ferroptosis Induction Atlas; https://deferrinda.fccps.ru) interface. **B** Bubble plots showing transcriptome‑ and proteome‑level GO terms enriched upon ferroptosis induction. Each bubble represents one GO term; bubble size reflects the number of significantly up‑regulated DEGs or DEPs contributing to the term, and color encodes the inducing condition, as indicated in the legend.

We examined ferroptosis-induced transcriptional changes by identifying differentially expressed genes (DEGs) and proteins (DEPs) at early (24 h) and late (48 h) time points relative to baseline (0 h) samples. To facilitate the exploration of identified ferroptotic regulators across both molecular levels, we developed a web atlas, Deferrinda (Differential Expression Ferroptosis Induction Atlas; https://deferrinda.fccps.ru/). In Deferrinda, users can select the ferroptosis induction method and timepoint, adjust thresholds for differential expression, protein abundance, and statistical significance, and explore ferroptosis-related regulators (Fig. 2A). The web atlas also enables downloading differential expression and gene ontology (GO) enrichment tables, visualizing volcano plots, and examining genes of interest within our datasets.

We carefully analyzed both shared and inducer-specific DEGs and DEPs and identified the following trends. Both chemical and genetic ferroptosis induction elicited modest responses at 24 h, which dramatically amplified by 48 h (Supplementary Table 1, Supplementary Table 2, Supplementary Fig. 2,3). Gene Ontology (GO) analysis revealed lipids and fatty acids metabolism enrichment across all ferroptotic conditions with cholesterol biosynthetic activation in GPX4 pathway inhibition by ML210 or Tam-induced KO (Fig. 2B).

### Two distinct pathways of ferroptosis induction: ATF4 and SREBF-mediated transcriptional regulation

**Fig. 3:**
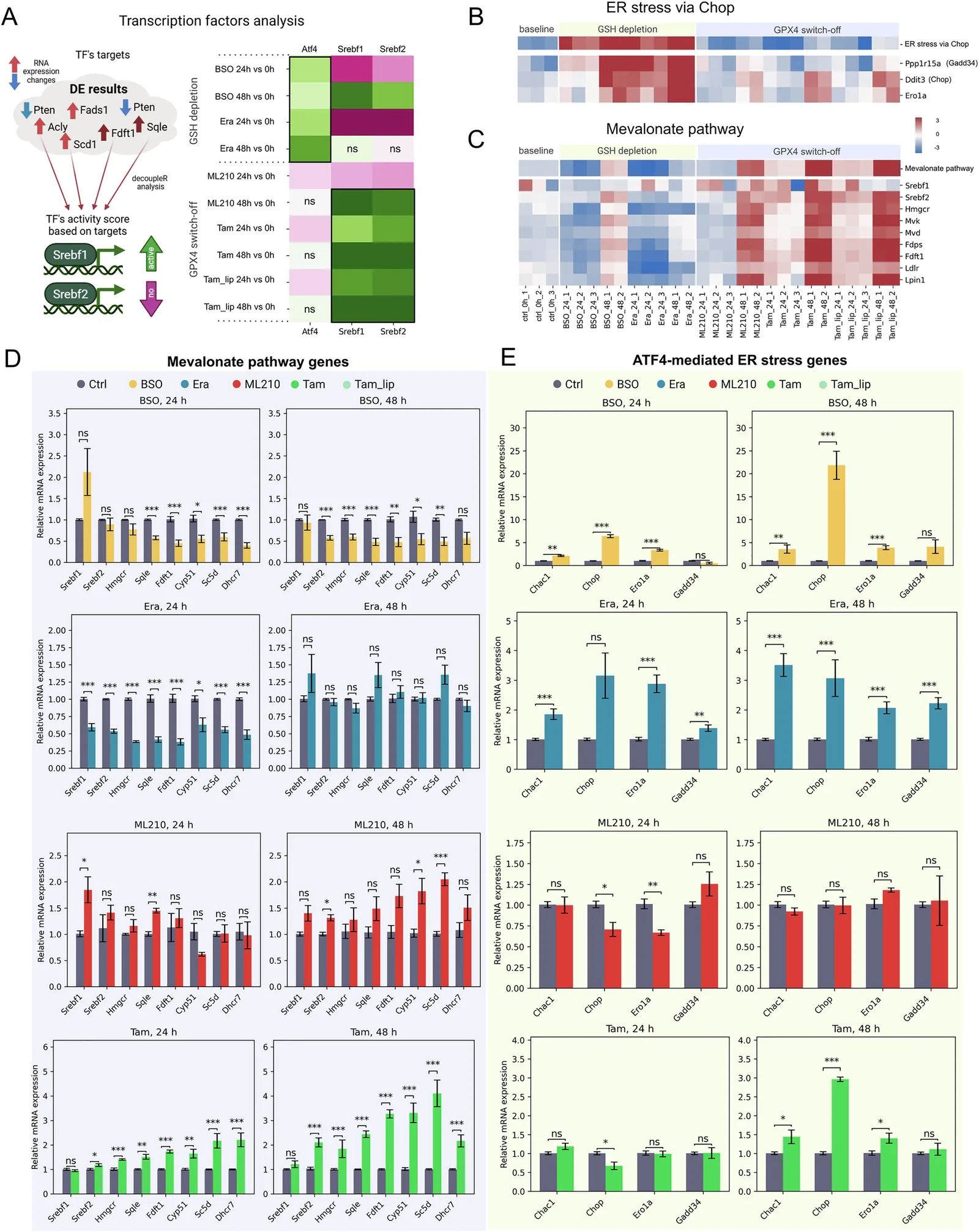
ATF4 and SREBF-mediated transcriptional regulation of ferroptosis. **A** Heatmap with summarized TF’s activity scores based on their gene targets. The x-axis shows the TFs that contributed most to the difference between glutathione depletion and GPX4 switch-off responses. Data has been clipped (-8, 8), ns stands for not significant TF’s scores. **B** Transcriptome analysis of ER stress. Upper heatmap - summarized gene-signature showing ER stress ssGSEA enrichment (*N* = 23 genes). Lower heatmap - scaled gene expression heatmap of key genes. **C** Transcriptome analysis of mevalonate pathway. Upper heatmap - gene signature (*N* = 16 genes) with key genes regulating mevalonate pathway activation. Lower heatmap - individual median-scaled gene expression heatmap of key genes. **D, E** RT-qPCR validation of ferroptosis-related gene expression changes. Bar graphs showing relative mRNA expression levels of selected MVA-associated (**D**) and ATF4-mediated ER stress genes (**E**) after treatment with BSO, Erastin, ML210, and Tamoxifen (Tam) compared to control. Data are presented as mean ± SEM from at least three independent experiments. Statistical significance was determined by Mann-Whitney test: *p < 0.05, **p < 0.01, ***p < 0.001; ns - not significant. Gene expression was normalized to ACTB.

We inferred transcription factor (TF) activity by analyzing expression changes in TF target genes, independent of the TF’s own expression levels. This analysis revealed pathway-specific regulatory signatures: ATF4 emerged as the predominantly activated transcription factor in BSO- and Era-treated samples, whereas GPX4-targeted conditions (ML210 and Tam) were characterized by activation of SREBF1/2 (Fig. 3A).

The predominant activation of ATF4 in xc⁻/GSH pathway inhibition conditions, but not in GPX4 knockout or inhibition conditions, is consistent with selective engagement of ER stress responses upon glutathione depletion rather than direct GPX4 loss. This pattern was supported by single-sample gene set enrichment analysis using a 23-gene ER stress signature, which showed significant enrichment in Era and BSO conditions (Fig. 3B, Supplementary Fig. 4E–H). Key ATF4 downstream targets were correspondingly upregulated, indicating transcriptional activation of this stress response pathway. These observations were further corroborated by RT-PCR validation of key ATF4 target genes across all ferroptosis induction conditions, which confirmed the condition-specific upregulation pattern identified in the RNA-seq dataset (Fig. 3E).

Conversely, GPX4-targeted ferroptosis (genetic knockout and chemical inhibition) was associated with robust upregulation of SREBF1/2 activity scores and coordinated induction of mevalonate pathway gene expression (Fig. 3A, C). While the mevalonate pathway is classically associated with ferroptosis protection through CoQ biosynthesis and selenocysteine tRNA maturation, our analysis revealed that these specific anti-ferroptotic branches remained inactive under our experimental conditions (Supplementary Fig. 5), suggesting that SREBFs activation may be associated with other mevalonate-dependent processes, such as cholesterol and fatty acid synthesis. Direct analysis of mevalonate pathway gene expression was consistent with these transcriptional patterns: all 16 MVA pathway genes were upregulated in GPX4 knockout samples and in late ML210-treated cells, while BSO and Era treated samples showed relatively reduced expression of these genes (Fig. 3C, Supplementary Fig. 4A-D). These patterns were independently validated by RT-PCR quantification of representative MVA pathway genes, confirming the RNA-seq-based observations (Fig. 3D). Collectively, these findings are consistent with GPX4 loss being preferentially associated with mevalonate pathway upregulation, whereas GSH/xc⁻ depletion is associated with its suppression - a reciprocal transcriptional relationship that may reflect fundamentally distinct cellular stress adaptation strategies.

### Independent datasets validate pathway-specific transcriptional signatures of ferroptosis induction

**Fig. 4:**
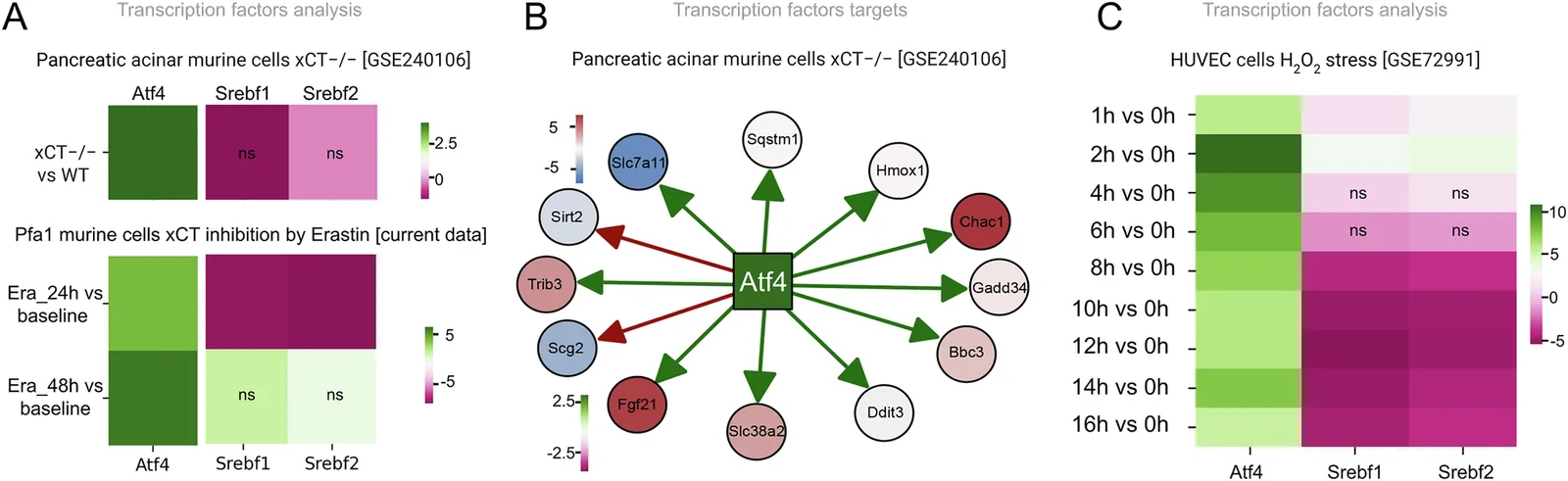
Transcription factors activity: validation on public datasets confirm Atf4 activation via GSH depletion ferroptosis induction. **A** Transcription factors activity analysis (decoupler, RNAseq) on knockout (KO) xCT pancreatic acinar murine cells [GSE240106] compared to erastin exposure on study data (PFA-1 cell line). ns stands for not significant TF’s activation. **B** Network of ATF4 TF’s targets on knock out xCT pancreatic acinar cells [GSE240106]. Color of targets stands for their expression level: red - high expression, blue - low expression. Сolor of the arrows indicates the activation (green) or deactivation (red) of the target under TF’s influence. Сolor of the rectangle indicates TF’s activity score. **C** Transcription factors activity analysis (decoupler, RNAseq) after H2O2 exposure on HUVECs (human umbilical vein endothelial cells) [GSE72991] at different time points.

To assess whether ATF4 activation is a reproducible feature of system xc⁻ disruption, we analyzed publicly available RNA-seq data from genetic xCT knockout in murine pancreatic acinar cells [12]. This independent dataset showed ATF4 activation patterns consistent with our observations, including upregulation of key target genes such as Chac1, Trib3, Bbc3 (Puma), and Fgf21, which were also elevated in our Era-treated samples at 48 h (Fig. 4A, B). This validation dataset also showed downregulation of SREBF1 and SREBF2 activity scores, consistent with the pattern we observed following Era treatment. These findings suggest that system xc⁻ disruption, whether through chemical inhibition or genetic knockout, is associated with ATF4-mediated stress response activation and concurrent suppression of SREBF1/2 activity - a transcriptional pattern that contrasts with GPX4 knockout-induced responses.

To explore potential overlap between ferroptosis-associated transcriptional changes and broader oxidative stress responses, we analyzed published transcriptomic data from long-term H₂O₂ exposure (1–16 h) in human umbilical vein endothelial cells (HUVECs) [13]. We observed ATF4 transcriptional program activation with peak activity at 2 h followed by gradual decline, while SREBF1/2 activity showed initial mild suppression at 4 h that progressed to more pronounced downregulation throughout the exposure period (Fig. 4C). These H₂O₂-induced transcriptional changes showed qualitative similarities to our BSO and Era treatment results, suggesting potential common transcriptional response elements between general oxidative stress and xc⁻/GSH axis disruption.

Together, these analyses indicate that ATF4 activation upon xc⁻/GSH disruption is observed across independent models and cell types. The qualitative overlap with H₂O₂-induced oxidative stress responses is consistent with glutathione depletion as a shared proximal trigger, while the contrasting SREBF1/2 response patterns between xc⁻/GSH inhibition and GPX4 knockout conditions support the existence of transcriptionally distinct ferroptosis induction pathways.

## Conclusions

**Fig. 5:**
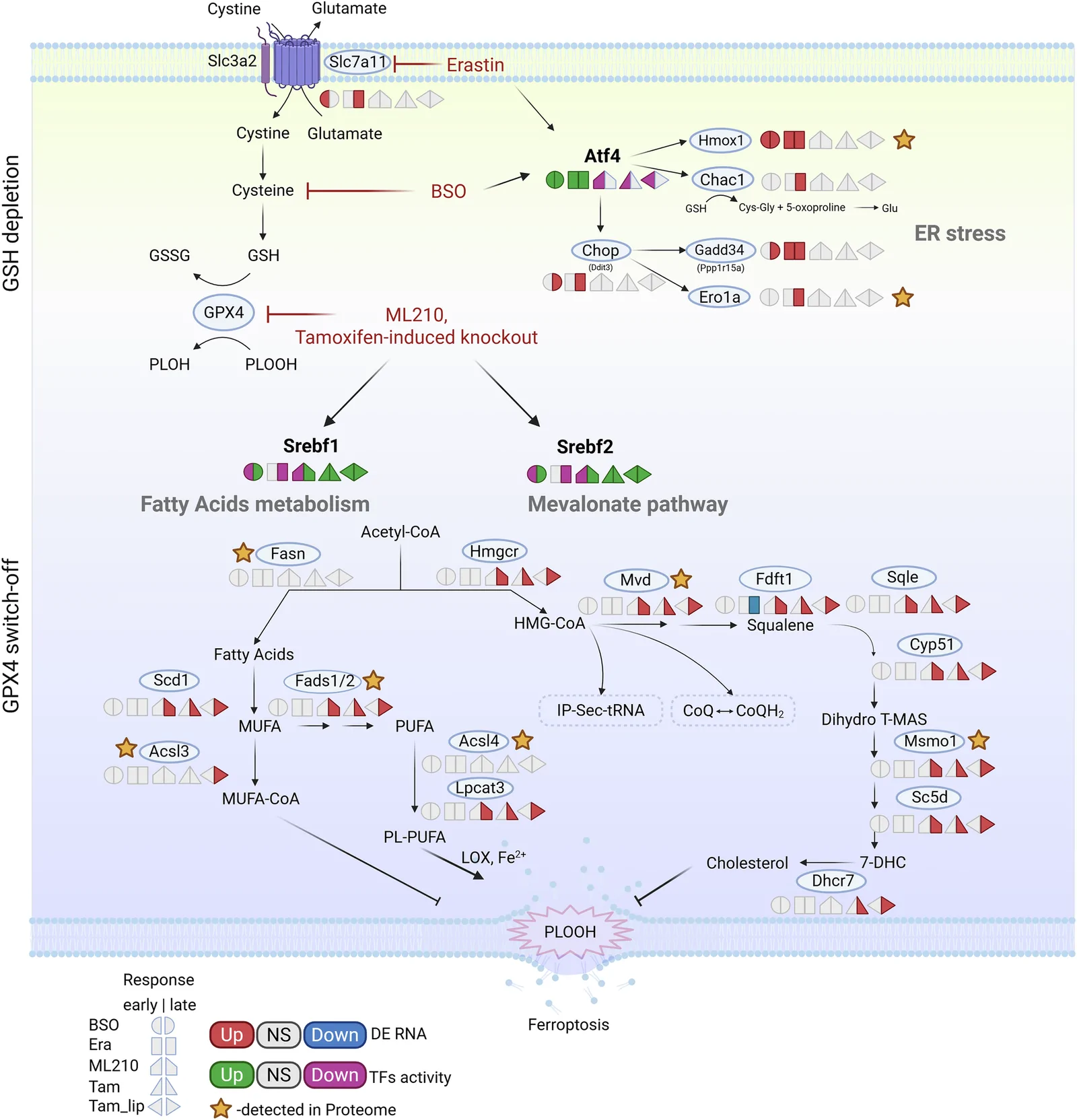
Dual ferroptosis regulation: ATF4-driven stress response versus SREBF-mediated metabolic reprogramming revealed by RNA and proteome sequencing in this study. Schematic depicting molecular pathways and key regulators of ferroptosis induction. Effects on glutathione supply and synthesis in yellow, GPX4 inhibition/knock-out in purple. Icon’s color represent a significant increase (red) or decrease (blue) on RNA level. The green-pink palette reflects the activity of transcription factors (green - activated TF, pink - deactivated TF). Star indicates detection at the proteome level. Created in BioRender. Abakumova, T. (2026) https://BioRender.com/1cy6ien.

Our comparative multi-omics analysis identifies two transcriptionally distinct response patterns associated with different ferroptosis induction strategies, converging on the central xc⁻/GSH/GPX4 regulatory axis (Fig. 5). The first pattern, observed upon system xc⁻/GSH pathway disruption, is characterized by preferential activation of ATF4 target genes consistent with an ER stress response directed at restoring amino acid homeostasis and antioxidant capacity. The second pattern, associated with GPX4 deficiency, is marked by upregulation of SREBF1/2 target genes and coordinated induction of the mevalonate pathway and lipid metabolic programs. These pathway-specific transcriptional signatures suggest that distinct ferroptosis induction strategies engage mechanistically divergent cellular stress responses - an observation that may have implications for how GSH-dependent and GPX4-dependent axes are considered in future therapeutic research. Both processed and raw data are publicly accessible and could serve as a valuable resource for comprehensive dynamic transcriptome and proteome mapping, facilitating future ferroptosis research. Differentially expressed ferroptotic regulators identified across all experimental conditions at both molecular levels are available for interactive exploration in Deferrinda web atlas (Differential Expression Ferroptosis Induction Atlas; https://deferrinda.fccps.ru/).

## Discussion

Our multi-omics analysis supports a view of ferroptosis as a pathway-dependent process associated with distinct transcriptional programs, rather than a uniform cell death mechanism. The pathway specificity we observe likely reflects evolutionarily distinct cellular crisis management systems. The ATF4-mediated response to glutathione depletion is consistent with an ancient amino acid starvation response co-opted to address oxidative stress through system xc⁻ upregulation and ER stress mitigation. Conversely, SREBF activation following GPX4 loss is consistent with a membrane-centric protection strategy, prioritizing lipid composition remodeling over antioxidant enzyme mobilization. This interpretation is consistent with the observation that GPX4 knockout conditions most closely resemble physiological ferroptosis triggers such as selenium deficiency, aging-related GPX4 decline, or copper-induced GPX4 aggregation - all scenarios where gradual GPX4 loss allows compensatory SREBF-mediated membrane stabilization.

GPX4 inactivation through tamoxifen and ML210 treatment activated a specific transcriptional response characterized by robust upregulation of SREBF1 and SREBF2 target genes, associated with coordinated induction of both the mevalonate (MVA) pathway and phospholipid biosynthesis. This metabolically-focused protective strategy is distinct from the ER stress-centered responses observed with system xc⁻ inhibition. The balance between polyunsaturated fatty acid incorporation, which promotes lipid peroxidation through genes such as ACSL4 and LPCAT3 [14, 15], and monounsaturated fatty acid synthesis, which provides ACSL3-dependent membrane protection [9], fundamentally determines cellular ferroptosis susceptibility. While MVA intermediates squalene and 7-dehydrocholesterol possess documented anti-ferroptotic properties [6, 8], our analysis revealed coordinated upregulation of both synthetic enzymes (FDFT1, SC5D) and downstream metabolizing enzymes (SQLE, DHCR7), indicating these protective intermediates are unlikely to accumulate significantly. We hypothesize that the ultimate MVA product, cholesterol, may confer ferroptosis resistance through membrane biophysical effects, as cholesterol incorporation can decrease membrane fluidity and permeability, potentially limiting lipid peroxidation propagation [16].

GSH depletion through system xc⁻ inhibition (Era) or γ-glutamylcysteine-synthetase blockade (BSO) consistently activated an ATF4-centred transcriptional program. In line with previous work showing PERK-eIF2α-ATF4-CHOP engagement during system xc⁻-driven ferroptosis [17, 18], we detected marked induction of canonical ATF4 targets - CHAC1, TRIB3 and SLC7A11. CHAC1 may further lower the residual GSH pool and thereby stabilize NRF2 [17], while TRIB3 could modulate ferroptosis either by enhancing SLC7A11 or repressing ALOXE3 [19, 20]. We also observed increased ERO1-α, a CHOP effector that has been proposed to reinforce SLC7A11 via IL-6/STAT3 signaling [21]. Together, these changes are consistent with an adaptive feedback loop that may temporarily oppose ferroptosis by boosting cystine import and redox capacity, even as continued GSH loss drives oxidative stress.

## Materials and methods

### Cell culture and ferroptosis induction

Pfa1 cells, 4-OH-Tam-inducible Gpx4^−/−^ immortalized mouse fibroblasts, were kindly provided by Dr. Marcus Conrad (Munich, Germany). Pfa1 cells were cultured in RPMI-1640 medium (PanEko, Russia) containing glucose (2 g/L), 2 mM GlutaMAX Supplement (Gibco, Thermo Fisher Scientific, USA), 10% fetal bovine serum (BioSera, France), and pen/strep (1%) (PanEko, Russia) at 37°C with 5% CO2 in a humidified incubator (NuAire, USA). All cell lines were routinely tested for mycoplasma contamination and confirmed negative.

For ferroptosis induction, 300,000 Pfa1 cells were seeded on 100 mm tissue-culture-treated culture dishes (#CLS430167, Corning, USA) and incubated at 37°C with 5% CO2 overnight. On the next day, cells were treated with or without: (i) 0.5 µM erastin (Era) (E7781, Sigma-Aldrich); (ii) 50 µM L-Buthionine-sulfoximine (BSO) (B2515, Sigma-Aldrich); (iii) 0.3 µM ML210 (SML0521, Sigma-Aldrich, USA); (iv) 1 μM (Z)-4-Hydroxytamoxifen (Tam) (H7904, Sigma-Aldrich); (v) 1 μM (Z)-4-Hydroxytamoxifen (H7904, Sigma-Aldrich) and 0.5 µM Liproxstatin-1 (SML-1414-25MG). The concentrations of the chemicals under scrutiny (Era, BSO, and ML210) were selected in order to correspond with the relative viability of cells as observed in the genetic experiment (Tam-GPX4-KO) (Data not shown). Cell collection for further analysis was performed 24 h and 48 h after treatment.

### Dual-parameter BODIPY 581/591 C11 / propidium iodide flow cytometry

Pfa1 cells (100,800) were seeded on 60 mm tissue-culture treated dishes and incubated for 1.5 h at 37°C with 5% CO2. After incubation, cells were treated with or without: (i) 0.5 µM erastin (E7781, Sigma-Aldrich); (ii) 50 µM L-Buthionine-sulfoximine (B2515, Sigma-Aldrich); (iii) 0.3 µM ML210 (SML0521, Sigma-Aldrich, USA); (iv) 1 μM (Z)-4-Hydroxytamoxifen (H7904, Sigma-Aldrich); (v) 1 μM (Z)-4-Hydroxytamoxifen (H7904, Sigma-Aldrich) and 0.5 µM Liproxstatin-1. 24 h and 48 h later, cells were treated with 1.5 μM BODIPY 581/591 C11 (Thermo Fisher) and with 1.5 μM Propidium iodide (Lumiprobe) for 1 h at 37°C with 5% CO2. Further cells were trypsinized, pelleted by centrifugation, resuspended in PBS (500 μL) and analyzed with a BD FACSCanto Flow Cytometer (488 laser for excitation, 530/30 band-pass filter for BODIPY signal; 488 laser for excitation, 695/40 nm band-pass filter for PI signal). At least 35,000 cells were analyzed per sample. Data analysis was performed using FlowJo software.

### Cell viability assay (MTT)

PFA1 cells were seeded into 96‑well plates at a density of 1500 cells per well in RPMI-1640 medium (PanEko, Russia) containing glucose (2 g/L), 2 mM GlutaMAX Supplement (Gibco, Thermo Fisher Scientific, USA), 10% fetal bovine serum (BioSera, France), and pen/strep (1%) (PanEko, Russia) at 37°C with 5% CO2 in a humidified incubator (NuAire, USA). After 1.5 h of incubation to allow cell attachment, ferroptosis inducers were added at the following final concentrations: 0.5 µM erastin (E7781, Sigma-Aldrich), 50µM L-Buthionine-sulfoximine (B2515, Sigma-Aldrich), 0.3 µM ML210 (SML0521, Sigma-Aldrich, USA), 1 μM (Z)-4-Hydroxytamoxifen (H7904, Sigma-Aldrich), 1 μM (Z)-4-Hydroxytamoxifen (H7904, Sigma-Aldrich) and 0.5 µM Liproxstatin-1. Control wells received an equivalent volume of vehicle (medium without drugs). At 24 h and 48 h post‑treatment, cell viability was assessed using the MTT (3‑(4,5‑dimethylthiazol‑2‑yl)‑2,5‑diphenyltetrazolium bromide) (PanEko, Russia) assay. MTT was dissolved in the cell culture medium at a concentration of 5 mg/mL, and 100 μL of this solution was added to each well. After 3 h of incubation at 37°C with 5% CO2 in a humidified incubator, the MTT‑containing medium was carefully removed, and 100 μL of dimethyl sulfoxide (DMSO) (PanEko, Russia) was added to each well to dissolve the formazan crystals. Optical density was measured at 570 nm using a Varioskan LUX Multimode Microplate Reader (Thermo Fisher Scientific, USA). For data analysis, the background signal was subtracted from all raw absorbance values. The background was defined as the average optical density measured in wells that received only cell culture medium with MTT, followed by the same removal and DMSO addition steps (no‑cell blanks). The corrected absorbance of each experimental well was then normalized to the average corrected absorbance of control cells (cultured without any ferroptosis inducer) and expressed as a percentage of viable cells relative to the control: ${cell\; viability}( \% )=\frac{{{OD}}_{{sample}}}{{{OD}}_{{control}}}* 100 \%$. All conditions were tested in six replicate wells per experiment and seven biological replicates. In the box‑and‑whisker plots, the boxes represent the interquartile range (IQR), with the line inside the box indicating the median, and the whiskers extending to the minimum and maximum non‑outlier values. All individual data points are overlaid as jittered dots to visualize the full distribution. Statistical analysis was performed using Kruskal–Wallis ANOVA; ** indicates *p*‑value < 0.01 and *** indicates *p*‑value < 0.001.

### RT-qPCR

RT-qPCR was performed using 5× qPCRmix-HS SYBR containing SYBR Green I (Evrogen, Russia) according to the manufacturer’s instructions. Reactions (25 µL total volume) contained 5 µL of 5× master mix, forward and reverse primers at a final concentration of 0.4 µM each, and 1 µL of cDNA template; nuclease-free water was added to volume. Primers were selected based on published data and the PrimerBank database. Amplification was carried out on a LightCycler 96 system (Roche) using the following cycling conditions: initial denaturation at 95°C for 300 s, followed by 45 cycles of 95°C for 25 s, 60°C for 20 s, and 72°C for 25 s. Relative gene expression was calculated using the 2^−ΔΔCt method. Ct values were normalized to the housekeeping gene β-actin, and ΔΔCt values were determined relative to the control group. Statistical significance between ferroptosis-induced and control samples was assessed using the Mann-Whitney U test.

### Proteome data

#### Extraction and preparation of proteins for MS analysis

The unmodified trifluoroethanol (TFE)-containing protocol [22] was employed for preparing the samples. Cells (~1 × 10^6) were washed three times with PBS. Subsequently, they were treated with 80% ice-cold methanol, scraped, incubated (1 h, −80°C), and centrifuged. The pellets were rinsed with cold acetone before next centrifugation and dried on air. For protein extraction, the pellets were resuspended via sonication in 120 μL of 50 mM ammonium bicarbonate (ABC) buffer, pH 8.0, combined with equal volume of TFE, then supplemented with 5 mM TCEP for reduction (1 h, 50°C), and 15 mM iodoacetamide (IAA) (1 h, RT). Afterwards, the samples were diluted fourfold with ABC buffer prior to addition of 2 µg of trypsin/LysC blend. The enzymatic digestion proceeded overnight at 37°C, and the reaction was quenched by acidification with formic acid (1%). Peptides were dried in a vacuum centrifuge (3 h, 45°C), redissolved, and quantified utilizing a BCA assay.

#### LC-MS/MS analysis

Liquid chromatography–mass spectrometry (LC-MS/MS) was carried out with a Q Exactive HF-X mass spectrometer (Thermo Scientific, Bremen, Germany) connected to an UltiMate 3000 nano-flow liquid chromatography setup (Thermo Scientific, Dreieic, Germany). Initially, one microgram of peptide mixture dissolved in Buffer C (composition described below) was injected on an Acclaim μ-Precolumn enrichment cartridge (Cat.#160454, Thermo Scientific, Vilnius, Lithuania), under isocratic flow of Buffer C (2% acetonitrile and 0.1% trifluoroacetic acid in MS-grade water) at a rate of 5 µL/min for five minutes in the ML210, BSO and Era experiments, or at a rate of 10 µL/min for 1,5 min in the Tam experiment. Peptides were then separated either on an Acclaim Pepmap 100 C18 analytical column (Cat.#164570, Thermo Scientific, Vilnius, Lithuania) in the Tam, ML210 and Era experiments or on an Acclaim Pepmap RSLC column (Cat.#164939, Thermo Scientific, Vilnius, Lithuania) in the BSO experiment in a binary gradient of Solvent A (0.1% formic acid in MS-grade water) and Solvent B (80% acetonitrile and 0.1% formic acid in MS-grade water), delivered at a constant flow rate of 0.3 µL/min (or 0,4 µL/min in the Tam experiment). In Era and ML210 experiments, the gradient consisted of the following steps: 4% B (5 min), from 4 to 33% B (100 min), from 33 to 45% B (30 min), from 45 to 95% B (1 min), 95% B (20 min), from 95 to 4% B (1 min), and 4% B (30 min). In the BSO experiment the gradient was as follows: 4% B (6 min), from 4 to 30% B (95 min), from 30 to 50% B (25 min), from 50 to 95% B (2 min), 95% B (15 min), from 95 to 4% B (2 min), and 4% B (20 min). In the Tam experiment the steps were: 2% B (1 min), 2 to 35% B (94 min), 35 to 99% B (4 min), 99% B (5 min), 99 to 2% B (2 min), 2% B (5 min).

The MS/MS analysis was carried out in positive ionization mode utilizing a NanoFlex ESI ion source. The source and MS parameters were as follows: in the Era, ML210 and BSO experiments emitter voltage, 2.3 kV; capillary temperature, 260°C; and in the Tam experiment emitter voltage, 2.1 kV; capillary temperature, 240°C. Panoramic scan: in the Era, ML210 and BSO experiments mass range, 350–1800 m/z; 60,000 resolution; in the Tam experiment mass range, 390–1500 m/z; 120,000 resolution. Tandem scan: dynamic mass range; 15,000 resolution. Precursor isolation window: in the Era, ML210 and BSO experiments 1.2 Da; in the Tam experiment - 1 Da. DDA mode was set to “top20”, the intensity cutoff limit for precursor selection was 44,000 in the Era, ML210 and BSO experiments, and 40,000 in the Tam experiment; NCE: 28 units in the Era, ML210 and BSO experiments, and 29 units in the Tam experiment. Ions with charge states from +1 to +6 in the Era, ML210 and BSO experiments, and from +2 to +4 in the Tam experiment were selected for MS2 analysis. In the Era, ML210 and BSO experiments accumulation times were set to 60 ms and 45 ms for precursor and product ions, respectively. In the Tam experiment accumulation times were set to 50 ms and 100 ms for precursor and product ions, respectively. In the Era, ML210 and BSO experiments the AGC values for precursor ions were 3*10^6^, and 1*10^5^ for fragment ions. In the Tam experiment the AGC values for precursor ions were 1*10^6^, and 2*10^5^ for product ions. All the precursor ions measured were dynamically excluded from the tandem MS analysis for 30 s in the Era, ML210 and BSO experiments, and for 20 s in the Tam experiment.

### Proteome data - bioinformatics analysis

Raw files were converted into *.mzML format using the msConvert console application [23]. Next, we used MSFragger 3.5 to align spectra to the protein reference with the following parameters: true precursor tolerance, 12 ppm; fragment mass tolerance, 50 mDa [24]. As a reference we used the standard mouse SwissProt proteome (UP000000589) augmented with standard contaminants of the Philosopher package [25] and with FBS -derived contaminants previously measured in our lab in FBS samples on the same instrument.

To exclude false-positive results, we performed target-decoy analysis on the samples with grouped technical replicates using peptideProphet [26], employing proteinProphet [27] for further statistical clarification. Label-free quantification was conducted by IonQuant 1.8 [28] with the match-between-run option turned on. Next, low covered proteins were filtered out if they were covered by fewer than two unique/razor peptides. The DEP package [29]. was used for visualization of the protein numbers. Detailed description of the data acquisition and processing protocol are described in [30, 31].

In total we processed 128 samples with 3-4 biological and 2-3 technical replicates for each condition, samples were splitted in four series. Control samples (baseline Pfa1 cells with no treatment) were included in each group of LS-MS/MS to mitigate batch effects. Due to the strong correlation between control samples we calculated linear normalization coefficients and normalized proteome signals between batches. Future acquisition of new data on similar techniques with normalization to control samples will allow correct comparison of these data with newly generated information.

### RNA sequencing and bioinformatician analysis

All conditions of ferroptosis induction (BSO, Era, ML210, Tam, Tam_lip) in 2 timepoints (24 h and 48 h) and control (0 h, no treatment) samples of Pfa1 cell line were prepared for RNA sequencing. RNA was extracted according to the manufacturer’s instructions using the Qiagen RNeasy Mini Kit (Qiagen). The quality of total RNA was evaluated using a Bioanalyzer 2100 (Agilent). The quantity and purity of RNA was estimated on a NanoPhotometer (Implen). 800 ng of RIN ≥ 7 of total RNA was used for library construction using a NEBNext® Poly(A) mRNA Magnetic Isolation Module and NEBNext® Ultra II™ Directional RNA Library Prep Kit for Illumina (New England Biolabs) according to the manufacturer’s instructions. The quality of libraries was verified using the Bioanalyzer 2100 (Agilent) and the yield was validated by qPCR. Libraries were then sequenced on a NovaSeq6000 (Illumina) with pair-end 61 bp reading.

The quality of the reads was verified using FastQC. For subsequent gene expression analysis, reads were aligned to the GENCODE M32 mouse reference genome by STAR [32] and quantified by FeatureCounts [33].

For differential analysis expression (DE) limma R package was used. Early and late samples were compared to the control (0 h, no treatment) independently. The significance (Adjusted *p*-value) and fold-change (FC) cutoffs for differentially expressed genes (DEGs) were Adj. *p*-value < 0.05 and |FC | > 1.5 respectively. Up- and down-regulated DEGs were subjected to GO analysis with clusterProfiler R package. To annotate ferroptosis drivers and suppressors we used the FerrDB V2 database [34].

For signature evaluation data were processed from raw FASTQ using Kallisto [35] and reference transcriptome GENCODE M32. Only protein coding transcripts were considered in the study and expression was quantified as transcripts per million (TPM) values and log2 normalized. Gene signatures were calculated as ssGSEA enrichment scores [36].

Gene signature MARCINIAK_ER_STRESS_RESPONSE_VIA_CHOP [37] with 23 genes was used to evaluate the enrichment of endoplasmic reticulum (ER) stress. Mevalonate synthesis pathway signature included 16 genes and was manually curated from [38–40].

Transcription factor activity was analyzed using the decoupleR program [41]. DecoupleR estimates the score of TF’s activity based on the expression of TF’s transcriptional gene-targets and their TF-gene interaction weight (positive weight means the activation; negative - deactivation). *P*-value was obtained by evaluating the two-sided survival function of the Student’s t-distribution and adjusted by Benjamini-Hochberg correction.

For public RNASeq datasets, raw reads were downloaded from GEO and SRA databases using the NCBI SRA Toolkit program. GSE240106 data with xCT knockout pancreatic acinar cells were calculated using the same pipeline. For GSE72991 data processing with human umbilical vein endothelial cells under H_2_O_2_ stress conditions we used human reference GENCODE human Release 43 (GRCh38.p13).

### Web application

The Deferrinda web atlas (https://deferrinda.fccps.ru/) was implemented in Python using the Streamlit framework (https://streamlit.io/) to provide an interactive browser-based interface for filtering and exploring differential expression results. Transcriptomic and proteomic differential expression tables were loaded and processed with pandas (https://pandas.pydata.org/) and NumPy (https://pypi.org/project/numpy/) for tabular data handling, reshaping, and threshold-based filtration of up- and down-regulated genes and proteins. Excel input files were read using openpyxl (https://openpyxl.readthedocs.io/), and interactive volcano plots and bar charts were generated with Plotly (https://plotly.com/python/). The input files are also available in supplementary materials as Supplementary Table 1 and Supplementary Table 2. Gene Ontology enrichment analysis was performed through the goatools (https://github.com/tanghaibao/goatools), using the *Mus musculus* annotation space to identify enriched GO Biological Process, Molecular Function, and Cellular Component terms among threshold-defined gene sets.

## Supplementary information

Supplementary

Supplementary Table 1

Supplementary Table 2

## Data Availability

RNASeq data generated during this study is available in SRA via project PRJNA1336868. Public RNASeq datasets were downloaded from SRA NCBI database with identifiers: GSE240106 for pancreatic acinar murine cells with xCT knockout, GSE72991 for human umbilical vein endothelial cells under H_2_O_2_ stress conditions. Proteomic data deposited at the PRoteomics IDEntifications (PRIDE) Archive database with the following dataset identifiers: BSO experiment - PXD041415, Erastin experiment - PXD041463; ML210 experiment - PXD041327; Tamoxifen - PXD040094.
