# Supplementary material for: Unlocking ferroptosis heterogeneity: ATF4 versus SREBF transcriptional programs
Source: Cell Death Discov. 2026 Jul 3;12:383. doi: 10.1038/s41420-026-03238-0 (PMC13601565; doi:10.1038/s41420-026-03238-0)
Supplement: Supplementary file 1 [file 41420_2026_3238_MOESM1_ESM.pdf]

Supplementary Information

Unlocking ferroptosis heterogeneity: ATF4 versus SREBF transcriptional programs

Mariia V. Barannikova[1,2,a], Valeriy K. Sulyagin[3,4,a], Dmitry A. Korzhenevskii[1], Varvara D. Starodubova[3], Maria A. Sorokina[1,3,5], Alexey M. Nesterenko[1], Guzel R. Gazizova[1,6], Victoria P. Sulyagina[1,4], Oleg A. Gusev[1,5,7], Elena I. Shagimardanova[5,8], Vsevolod V. Belousov[1,3], Olga M. Kudryashova[1,3,*] & Arina G. Shokhina[1,3,**]

1 - Federal Center of Brain Research and Neurotechnologies, Federal Medical Biological Agency, 117997, Moscow, Russia

2 - Moscow Center for Advanced Studies, 20, Kulakova Str., 123592, Moscow, Russia

3 - Pirogov Russian National Research Medical University, 117997, Moscow, Russia

4 - Faculty of Biology, Lomonosov Moscow State University, 119234 Moscow, Russia

5 - LIFT, Life Improvement by Future Technologies Institute, 121205 Moscow, Russia

6 - Regulatory Genomics Research Center, Institute of fundamental medicine and biology, Kazan Federal University, Kazan, Russia

7 – Intractable Disease Research Center, Juntendo University, 113-8421, Tokyo, Japan

8 - Genomics and Bioimaging Core Facility, Moscow 121205, Russia

Supplementary figures:

Supplementary Figure 1. Ferroptosis induction and liproxstatin-1 rescue in the tamoxifen-induced GPX4 knockout model assessed by dual-parameter BODIPY 581/591 C11 / propidium iodide flow cytometry.
Supplementary Figure 2. Venn Diagrams for up-regulated DE genes and proteins.
Supplementary Figure 3. Venn Diagrams for down-regulated DE genes and proteins.
Supplementary Figure 4. ER-stress and MVA-pathway signatures
Supplementary Figure 5. Verification of Sec-tRNA and CoQ synthesis under the mevalonate pathway activation

Supplementary tables:

Supplementary Table1 - Differentially expressed genes RNAseq. (Uploaded as a separate .xlsx file).
Supplementary Table2 - Differentially expressed genes Proteome. (Uploaded as a separate .xlsx file).

Supplementary Figure 1. Ferroptosis induction and liproxstatin-1 rescue in the tamoxifen-induced GPX4 knockout model assessed by dual-parameter BODIPY 581/591 C11 / propidium iodide flow cytometry.

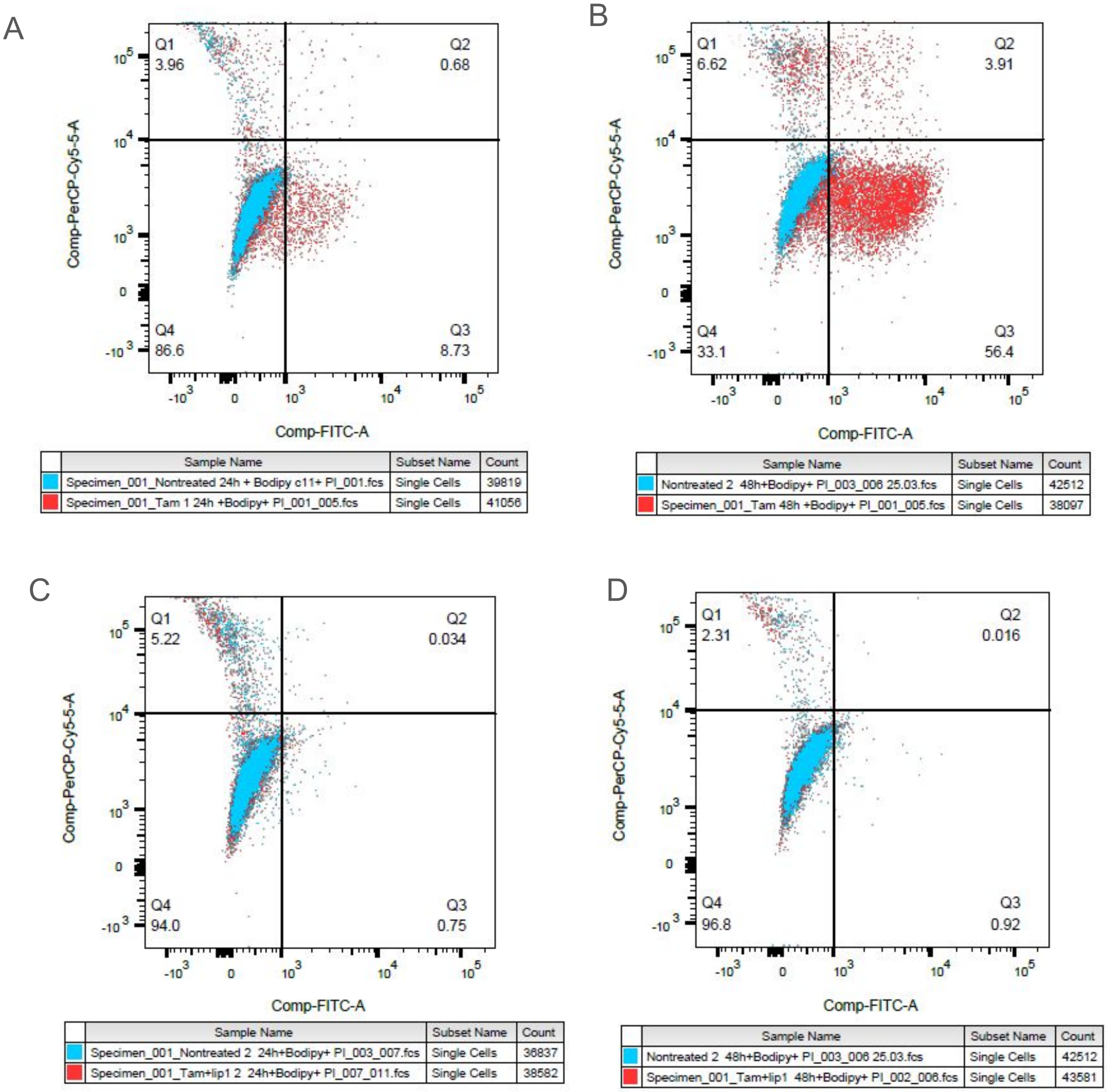

Supplementary Figure 1. Ferroptosis induction and liproxstatin-1 rescue in the tamoxifen-induced GPX4 knockout model assessed by dual-parameter BODIPY 581/591 C11 / propidium iodide flow cytometry. Representative dot plots showing BODIPY 581/591 C11 oxidation (x-axis, FITC channel; indicator of lipid peroxidation) and propidium iodide (PI) positivity (y-axis, PerCP-Cy5.5 channel; indicator of membrane integrity loss) in Pfa1 cells. Blue dots represent untreated control cells; red dots represent treated cells. Quadrant gates: Q2 (upper right) - BODIPY+/PI+ cells; Q3 (lower right) - BODIPY+/PI$^-$ cells (viable cells with active lipid peroxidation); Q1 (upper left) - PI+/BODIPY$^-$ cells; Q4 (lower left) - double-negative live cells. Numbers indicate percentage of gated single cells in each quadrant. (A) Untreated control vs. tamoxifen-treated cells (Tam) at 24h. (B) Untreated control vs. tamoxifen-treated cells (Tam) at 48h. (C) Untreated control vs. tamoxifen + liproxstatin-1 co-treated cells (Tam+Lip1) at 24h. (D) Untreated control vs. tamoxifen + liproxstatin-1 co-treated cells (Tam+Lip1) at 48h. At least 35,000 single cells were analyzed per sample. Representative plots from one of ≥3 independent biological replicates are shown. Statistical analysis of pooled replicate data is presented in Figure 1E.

Supplementary Figure2. Venn Diagrams for up-regulated DE genes and proteins.

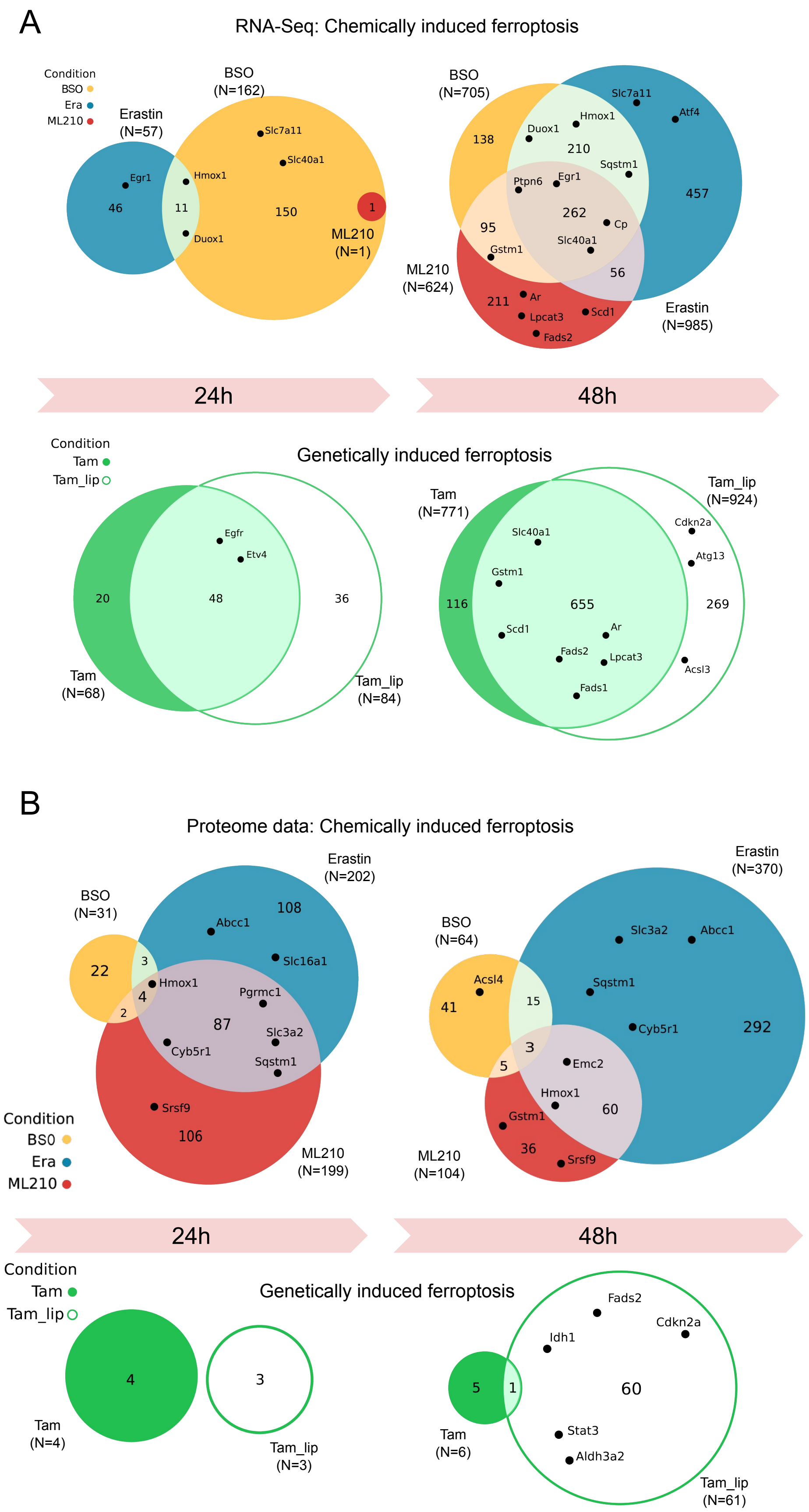

Supplementary Figure 2. Venn diagrams for up-regulated DE genes and proteins.

A Venn diagrams of up-regulated RNAseq differentially expressed genes (DEGs) (24 and 48 h) in comparison with control (0h). Upper panel - chemical inductors (BSO, Erastin, ML210). Lower panel - genetical inductors (Tam, Tam_lip). Some known ferroptoic regulators are highlighted. **B** Venn diagrams of up-regulated DE proteins (24 and 48 h) in comparison with control (0h). Upper panel - chemical inductors (BSO, Erastin, ML210). Lower panel - genetical inductors (Tam, Tam_lip). Some known ferroptoic regulators are highlighted.

Supplementary Figure3. Venn Diagrams for down-regulated DE genes and proteins.

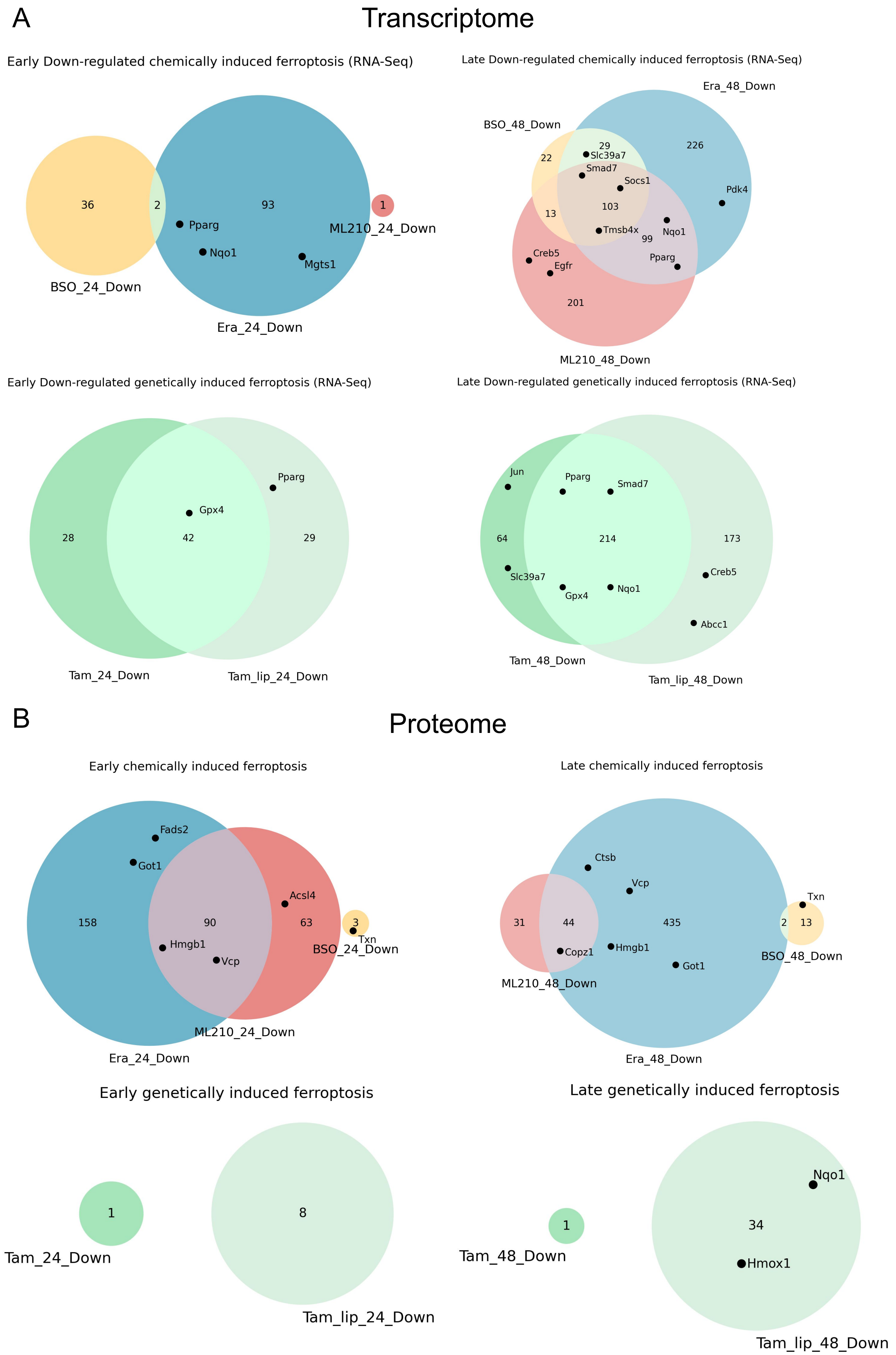

Supplementary Figure3. Venn Diagrams for down-regulated DE genes and proteins.

A Venn diagrams of down-regulated RNAseq differential expression genes (DEGs) (24 and 48h) in comparison with control. Upper panel - chemical inductors (BSO, Erastin, ML210). Lower panel - genetical inductors (Tam, Tam_lip). Some known ferroptoic regulators are highlighted. **B** Venn diagrams of down-regulated differential expression proteins (DEPs) (24 and 48h) in comparison with control. Upper panel - chemical inductors (BSO, Erastin, ML210). Lower panel - genetical inductors (Tam, Tam_lip). Some known ferroptoic regulators are highlighted.

Supplementary Figure4. ER-stress and MVA-pathway signatures.

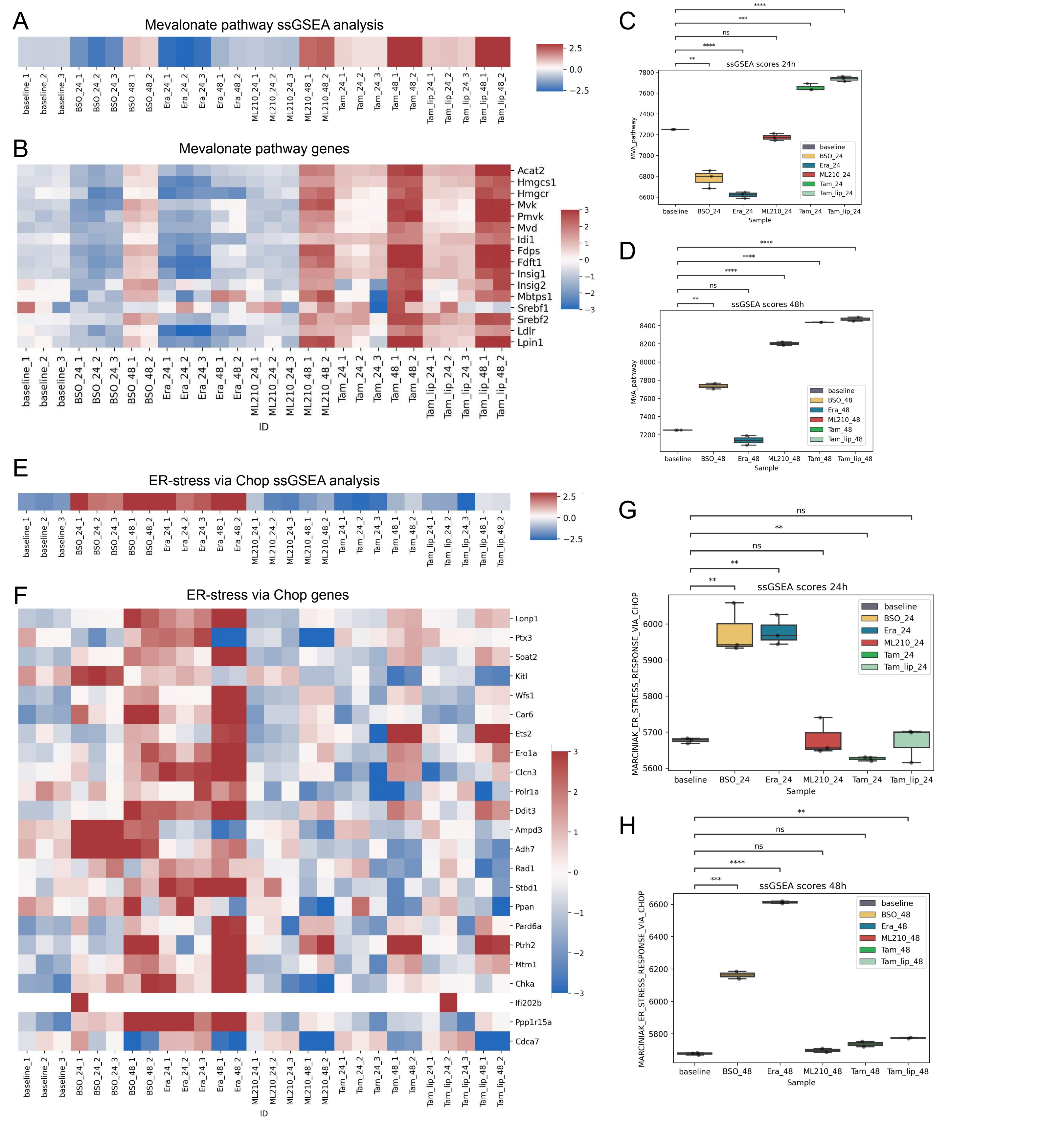

Supplementary Figure4. ER-stress and MVA-pathway signatures

A Transcriptome analysis of mevalonate pathway. ssGSEA of manually curated gene signature (N=16 genes). **B** Individual gene expression heatmap of key mevalonate pathway genes. **C-D** Boxplots for ssGSEA mevalonate pathway enrichment scores calculated by t-test independent samples with Bonferroni correction at 24h (C) and 48h (D). **E** Transcriptome analysis of ER stress. ssGSEA of msigDB gene signature showing ER stress activation (N=23 genes, MARCINIAK_ER_STRESS_RESPONSE_VIA_CHOP (DOI: 10.1101/gad.1250704). **F** ER-stress via Chop gene expression heatmap. **G-H** Boxplots for ssGSEA ER-stress via Chop enrichment scores calculated by t-test independent samples with Bonferroni correction at 24h (G) and 48h (H).
p-value annotation legend: ns: 5.00e-02 < p <= 1.00e+00, *: 1.00e-02 < p <= 5.00e-02, **: 1.00e-03 < p <= 1.00e-02, ***: 1.00e-04 < p <= 1.00e-03, ****: p <= 1.00e-04

Supplementary Figure5. Verification of Sec-tRNA and CoQ synthesis under the mevalonate pathway activation.

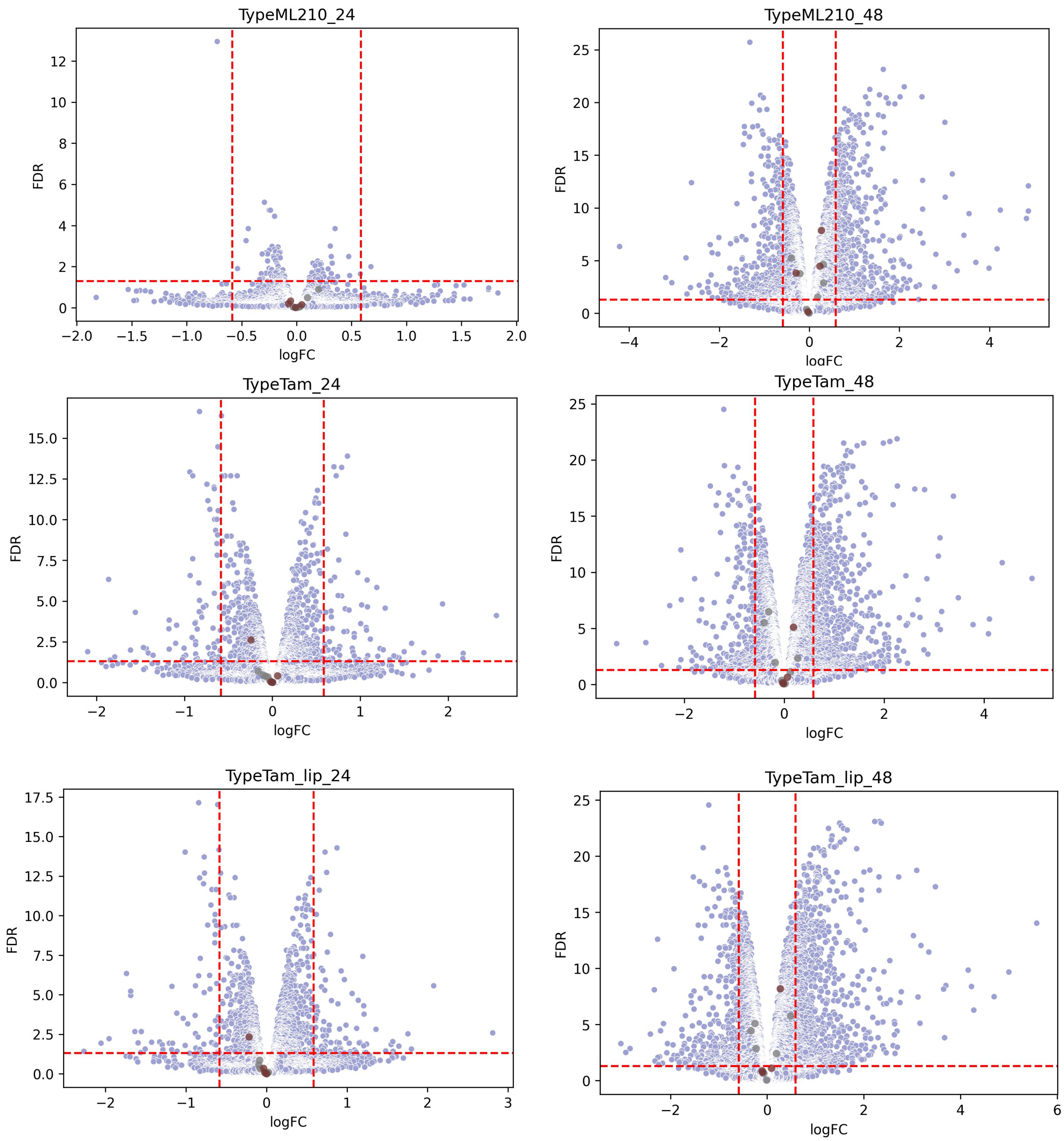

Supplementary Figure5. Verification of Sec-tRNA and CoQ synthesis under the mevalonate pathway activation. Differential expression (RNA-Seq) volcano plots after ferroptosis induction via GPX4 switch-off (ML210, Tam, Tam_lip). Grey color stands for CoQ synthesis genes (CoQ2, CoQ3, CoQ5, CoQ6, CoQ7, CoQ10a, CoQ10b), brown color stands for Sec-tRNA synthesis genes (Sars, Sepsecs, Sec, Sephs2, Trit1).
